# Identifying Talent in Youth Sport: A Novel Methodology Using Higher-Dimensional Analysis

**DOI:** 10.1371/journal.pone.0155047

**Published:** 2016-05-25

**Authors:** Kevin Till, Ben L. Jones, Stephen Cobley, David Morley, John O'Hara, Chris Chapman, Carlton Cooke, Clive B. Beggs

**Affiliations:** 1 Institute for Sport, Physical Activity and Leisure, Leeds Beckett University, Leeds, West Yorkshire, United Kingdom; 2 Discipline of Exercise & Sport Science, Faculty of Health Sciences, The University of Sydney, New South Wales, Australia; 3 Liverpool John Moores University, Liverpool, United Kingdom; 4 Sports Coach UK, Leeds, United Kingdom; 5 Leeds Trinity University, Leeds, United Kingdom; 6 Buffalo Neuroimaging Analysis Center, Department of Neurology, School of Medicine and Biomedical Sciences, University at Buffalo, Buffalo, NY, United States of America; Research Center for Sports Sciences, Health and Human Development (CIDESD), University of Trás-os-Montes e Alto Douro, Vila Real, Portugal, PORTUGAL

## Abstract

Prediction of adult performance from early age talent identification in sport remains difficult. Talent identification research has generally been performed using univariate analysis, which ignores multivariate relationships. To address this issue, this study used a novel higher-dimensional model to orthogonalize multivariate anthropometric and fitness data from junior rugby league players, with the aim of differentiating future career attainment. Anthropometric and fitness data from 257 Under-15 rugby league players was collected. Players were grouped retrospectively according to their future career attainment (i.e., amateur, academy, professional). Players were blindly and randomly divided into an exploratory (n = 165) and validation dataset (n = 92). The exploratory dataset was used to develop and optimize a novel higher-dimensional model, which combined singular value decomposition (SVD) with receiver operating characteristic analysis. Once optimized, the model was tested using the validation dataset. SVD analysis revealed 60 m sprint and agility 505 performance were the most influential characteristics in distinguishing future professional players from amateur and academy players. The exploratory dataset model was able to distinguish between future amateur and professional players with a high degree of accuracy (sensitivity = 85.7%, specificity = 71.1%; *p*<0.001), although it could not distinguish between future professional and academy players. The validation dataset model was able to distinguish future professionals from the rest with reasonable accuracy (sensitivity = 83.3%, specificity = 63.8%; *p* = 0.003). Through the use of SVD analysis it was possible to objectively identify criteria to distinguish future career attainment with a sensitivity over 80% using anthropometric and fitness data alone. As such, this suggests that SVD analysis may be a useful analysis tool for research and practice within talent identification.

## Introduction

Research in the talent identification (TID) of athletes within sport science has been of specific interest for approximately the last 15 years [1, 2]. TID is defined as the process of recognising current participants, at an early stage in their development, who have the potential to excel in a particular sport in adulthood [2, 3]. Many national governing bodies and professional clubs now invest considerable resources into the TID process in the hope of identifying the future stars of professional sport. Traditionally, TID research has attempted to differentiate uni- and multi-dimensional characteristics and qualities between elite, sub-elite and non-elite players using cross-sectional research designs (e.g., [4–7]) whereby young athletes are compared at specific time-points in order to identify player characteristics that may help predict future performance in adulthood [8].

Although TID has been of interest in recent years, there are limitations associated with many of the research designs used within this field. Firstly, given that the development of sporting talent is inherently multi-dimensional, influenced by numerous physical, technical, tactical and psychological factors [9], it would appear preferable to adopt a multi-dimensional approach when investigating TID in sport. However, much TID research is limited by its uni-dimensional approach [1–3]. Secondly, it is assumed that players’ current performance capabilities within junior populations can help predict potential success in adulthood [3]. Instead, a more appropriate method may be to retrospectively, or prospectively, track player characteristics into adulthood in order to better understand the factors that contribute to future performance. Recent studies in rugby league [10–12] and soccer [13–15] have used such longitudinal tracking designs to retrospectively compare player characteristics at junior ages (e.g., Under 15) with their future career attainment level (i.e., amateur, professional). For example, recently Till and colleagues [10] tracked junior rugby league players at Under 13, 14 and 15 age categories into adulthood and demonstrated anthropometry and fitness measures at junior levels had a significant impact upon future career attainment. Such studies have therefore advanced TID knowledge in relation to understanding player characteristics that may influence future adult performance.

A third limitation with traditional TID research, is that although the datasets can be very large, often containing many variables, the standard statistical analysis techniques used (e.g., t-tests, analysis of variance [ANOVA]) tend to ignore the multivariate aspects of the data, instead focusing on the identification of single variables to differentiate between performance levels. However, when dealing with datasets containing a large number of variables, many of which may be correlated, it is often difficult to discriminate between sub-groups within datasets using standard univariate techniques. Techniques such as multivariate analysis of variance (MANOVA) can enhance standard univariate analysis but they are limited when applied to sports performance data, due to the fact that the datasets used are often highly correlated, resulting in multicollinearity problems. In addition, it is not possible to visualise relationships within the data, or differences between comparative groups when using a MANOVA. However, through the use of higher-dimensional analysis techniques, such as singular value decomposition (SVD [16]) it is possible to overcome any multicollinearity problems and capture most of the variance in the data using just a few orthogonal variables, thus enabling differences between sub-groups to be more easily identified [17]. Such an approach, also allows differences between groups to be graphically presented, something that can be difficult to achieve when dealing with datasets containing a large number of variables.

Although SVD, a dimension reduction technique closely related to principal component analysis (PCA), has been used extensively in other disciplines (e.g., neuroimaging [18]), its use within sport science has to date been limited, with its applicability to TID, largely overlooked. We therefore undertook a retrospective study, with the aim of developing a higher-dimensional model to evaluate the extent to which long-term career attainment can be predicted using anthropometric and fitness data collected from junior (Under 15) rugby league players. This involved the development of a novel linear algebra methodology, coupling a SVD model with receiver operating characteristic (ROC) analysis to discriminate between groups of players, which we validated using a randomly selected subset of data. Because this model contained several novel methodological innovations, much of our paper is devoted to the methodology used in its development, as well as discussing its wider applicability to TID in sport and therefore develops upon previous research studies retrospectively tracking TID and anthropometric and fitness measures in rugby league [e.g., 10, 11].

## Materials and Methods

### Participants

Between 2005 and 2007 the Rugby Football League (RFL) in the United Kingdom (UK) operated a talent identification and development programme named the Player Performance Pathway (PPP; see [19] for more information). Between 2005 and 2007, 257 representative Under 15 rugby league players were selected to the RFL's PPP and so are the participants in the current study. By July 2008, PPP players were either: (a) selected to join a professional rugby league club's academy; (b) continued to play amateur rugby league; or (c) no longer participating in the game. Through training and competing within an academy, players were then potentially able to progress into playing adult professional rugby league within the UK Super League. Therefore, for the purposes of this study, players were divided into three career attainment levels for comparison, (1) 'amateur' (n = 98) not selected to an academy squad in 2008; (2) 'academy' (n = 123) selected to a professional rugby academy but did not play professional Super League; and (3) 'professional' (n = 36) played professional Super League by the end of the 2014 season, as used in previous research [10, 11]. Ethical approval was granted by Leeds Beckett University Ethics Committee with written assent provided by participants and written consent provided by parents/guardians.

### Protocols

All PPP players undertook an annual anthropometric and fitness assessment in July between 2005 and 2007. The protocol included standard anthropometry (height, sitting height, body mass, sum of 4 skinfolds), maturation (age at peak height velocity; PHV) and fitness (lower and upper body power, speed, change of direction speed, estimated VO_2max_) assessments for each participant. Assessments were undertaken at the same time of day (i.e., anthropometry pre breakfast and fitness early evening) and in the order described below. Prior to fitness testing, a standardized warm-up was performed and all players received full instructions. Intraclass correlation coefficients and typical error measurements for each measure are presented in previous research [6, 19] and all measurement reliability and objectivity conformed to published expectations [20].

Height and sitting height were measured using a Seca Alpha stadiometer, to the nearest 0.1 cm. Body mass was measured using calibrated Seca Alpha (model 770) scales, to the nearest 0.1 kg. Harpenden skinfold callipers (British Indicators, UK) were used to measure four skinfold thicknesses following the Hawes & Martin [21] procedures. To measure maturity status, an age at peak height velocity (PHV) prediction equation was used [22]. The 95% confidence interval associated with this equation for boys is ±1.18 years [22]. Years from PHV were calculated for each participant by subtracting age at PHV from chronological age.

The vertical jump test was used to assess lower body power via a Takei vertical jump metre (Takei Scientific Instruments Co. Ltd, Japan). A counter-movement jump with hands positioned on hips was used. Jump height was measured to the nearest cm with the highest value recorded during three attempts separated by 60 s rest [23]. A 2 kg medicine ball (Max Grip, China) chest throw was used to measure upper body power [24]. Participants were instructed to throw the ball horizontally as far as possible while seated with their backs against a wall. Distance was measured to the nearest 0.1 cm from the wall to where the ball landed from the best of three attempts separated by 60 s rest. Timing gates (Brower Timing Systems, IR Emit, USA) assessed sprint performance at 10 m and 60 m. Times were recorded to the nearest 0.01 s, with the best time recorded during 3 trials used for the sprint measurement, which were separated by 3 minutes rest. The agility 505 test assessed change of direction speed [25]. Participants started 15 m from a turning point with timing gates positioned 10 m from the start point. Players accelerated from the starting point, through the timing gates, turned on the 15 m line and ran as quickly as possible back through the gates. Three attempts were performed on each foot with times recorded to the nearest 0.01 s which were separated by 3 minutes rest. The multistage fitness test was used to assess estimated VO_2max_ [26]. Using a pre-recorded multistage fitness test compact disc, players were required to shuttle run 20 m keeping to a series of beeps. Player’s running speed increased progressively until they reached volitional exhaustion. Regression equations were used to estimate VO_2max_ from the level reached during the multistage fitness test.

### Statistical Analysis

**Fig 1:**
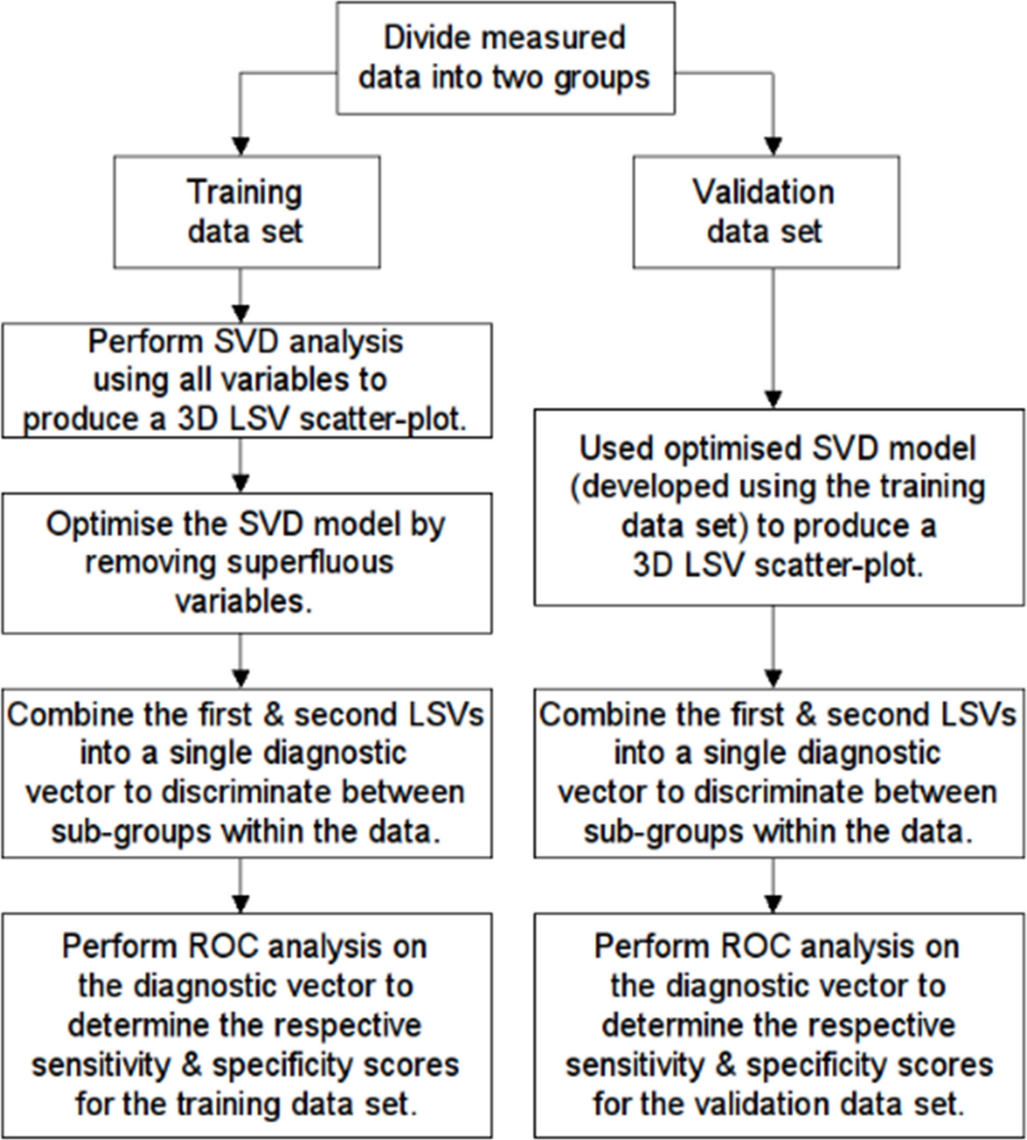
Schematic diagram of higher-dimensional analysis methodology employed in the study. (SVD = singular value decomposition; LSV = left singular vector).

Statistical analysis was undertaken using a combination of in-house algorithms written in Matlab (Math-Works, Natick, MA) and R (Open source statistical software). Fig 1 provides a schematic overview of the data analysis methodology undertaken.

In keeping with accepted statistical machine learning practice [27], participants were blindly divided, using a random number sampling algorithm, into two datasets: an exploratory dataset used to develop the metric model (n = 165; 64 amateurs, 80 academy, 21 professionals), and a validation dataset used to validate the model (n = 92; 34 amateurs, 43 academy, 15 professionals). This approach was adopted because it allowed us to mimic the situation where the findings of an exploratory dataset are confirmed in an independent follow-up study. The validation dataset was therefore deliberately blinded and used only to confirm the validity of the optimized higher-dimensional model, which was developed using the exploratory dataset. Care was taken to ensure that the amateur, academy and professional cohorts were represented in similar proportions in both datasets.

Next, univariate analysis of the data between the exploratory and validation datasets, and between the career attainment levels was undertaken using a combination of Student’s t-test (two-tailed) and a one-way ANOVA. Values of p<0.05 were considered statistically significant. The development of the higher-dimensional model was undertaken using SVD applied to the exploratory dataset (see Appendix). SVD was used to compute the first and second left singular vectors (LSVs) that accounted for most of the variance in the data. These were then plotted against each other enabling cluster analysis to be performed. Optimization of the model was done by excluding variables from the analysis that were deemed superfluous. This involved repeating the SVD analysis, systematically omitting each variable in turn and recording the impact of this on the Euclidean distance between the centroids of the amateur and professional player clusters. Variables that when omitted, increased the Euclidean distance, or had minimal effect, were deemed unhelpful and excluded from the model.

Once the model had been optimized, the first and second left singular vectors (LSVs; the LSVs which account for most of the variance in the data) were combined into a single vector, which was then rotated through 45° to produce a diagnostic vector (see Appendix). This diagnostic vector was then combined with a binary outcome classifier in a matrix and subjected to ROC analysis using a bespoke algorithm [28]. This enabled the optimum discriminating metric between the various sub-groups within the dataset to be identified and the calculation of the respective sensitivity and specificity (%) scores. Values of *p*<0.05 were considered statistically significant for the ROC analysis using a one-tailed test. Having developed and optimized the model using the exploratory dataset, the efficacy of the model was then evaluated using the validation dataset, which was used to calculate the respective sensitivity and specificity scores. Because the diagnostic vector was a weighted linear combination of the component variables, it was also possible to assess the relative contribution of the respective variables to the variance of the data system.

## Results

The results presented below reflect the order in which the higher-dimensional analysis was undertaken. This involved developing and optimizing a SVD model using the exploratory dataset and then testing its efficacy against the validation dataset.

### Between-group statistical results

Table 1 shows the exploratory and validation datasets. Univariate analysis revealed no significant differences between the two groups for any of the variables, except agility 505 right, which was significantly faster (*p* = 0.049) in the validation group.

**Table 1:** Univariate analysis results for the exploratory and validation datasets.

	Exploratory (n = 165)	Validation (n = 92)	P	Cohen’s d
Age (years)	15.57 ± 0.26	15.55 ± 0.28	0.570	0.075
Years from PHV (years)	1.82 ± 0.61	1.84 ± 0.56	0.850	-0.024
Height (cm)	177.1 ± 6.8	178.4 ± 5.8	0.115	-0.196
Body mass (kg)	74.7 ± 10.9	76.9 ± 11.3	0.143	-0.203
Sum of Skinfolds (mm)	40.1 ± 15.6	42.4 ± 16.7	0.281	-0.143
Vertical Jump (cm)	39.7 ± 5.6	38.9 ± 5.4	0.288	0.139
Medicine Ball Throw (m)	5.8 ± 0.8	5.8 ± 0.8	0.541	-0.080
10 m Sprint (s)	1.84 ± 0.16	1.88 ± 0.16	0.129	-0.205
60 m Sprint (s)	8.20 ± 0.41	8.22 ± 0.39	0.744	-0.046
Agility 505 right (s)	2.48 ± 0.16	2.44 ± 0.13	0.049	0.249
Agility 505 left (s)	2.51 ± 0.14	2.47 ± 0.13	0.067	0.241
Estimated VO_2max_ (ml.kg^-1^.min^-1^)	50.6 ± 4.8	50.2 ± 4.2	0.555	0.077

### Exploratory group results

Table 2 presents the anthropometric and fitness characteristics of the amateur, academy and professional players for the exploratory group. The one-way ANOVA revealed no significant differences between the amateur, academy and professional sub-groups for any of the variables, except 60 m sprint, which was significantly faster in the professional cohort (*p* = 0.023).

**Table 2:** Descriptive statistics and ANOVA results for the amateur, academy and professional sub-groups in the exploratory dataset.

	Amateurs (n = 64)	Academy (n = 80)	Professional (n = 21)	P
Years from PHV (years)	1.86 ± 0.60	1.77 ± 0.61	1.93 ± 0.64	0.478
Height (cm)	176.5 ± 6.7	176.9 ± 6.7	179.9 ± 7.1	0.154
Body mass (kg)	74.8 ± 12.1	74.0 ± 10.4	77.0 ± 9.5	0.509
Sum of Skinfolds (mm)	42.5 ± 15.4	38.8 ± 16.4	38.2 ± 11.9	0.284
Vertical Jump (cm)	39.6 ± 4.7	39.7 ± 5.9	40.0 ± 7.3	0.960
Medicine Ball Throw (m)	5.9 ± 0.7	5.7 ± 0.8	5.6 ± 1.2	0.412
10 m Sprint (s)	1.86 ± 0.16	1.85 ± 0.16	1.78 ± 0.13	0.109
60 m Sprint (s)	8.30 ± 0.46	8.15 ± 0.38	8.07 ± 0.24	0.023
Agility 505 right (s)	2.50 ± 0.16	2.47 ± 0.16	2.44 ± 0.17	0.297
Agility 505 left (s)	2.52 ± 0.12	2.51 ± 0.15	2.47 ± 0.14	0.439
Estimated VO_2max_ (ml.kg^-1^.min^-1^)	49.3 ± 5.5	51.2 ± 4.2	51.7 ± 3.8	0.065

Table 3 presents the results of the SVD analysis using all the variables in the exploratory dataset. This shows the linear coefficients of the respective variables in the first and second LSVs, together with percentage change in Euclidean distance between the centroids of the amateur and professional clusters when the respective variables are excluded from the SVD model. This reveals that the first LSV (LSV1) accounts for 37.4% of the variance in the data, while the second LSV (LSV2) accounts for a further 17.0%. Table 3 shows that the variables: height, body mass, agility 505 right, and 60 m sprint appear to strongly influence the Euclidean distance between the clusters, while the sum of 4 skinfolds, vertical jump and medicine ball throw either have a minimal or an adverse effect.

**Table 3:** Linear and rotated coefficients derived from SVD analysis of all the variables in the exploratory dataset.

	LSV1 coefficients	LSV2 coefficients	%Change in 2D Euclidean distance
Years from PHV	-0.0171	0.0291	-7.0%
Height	-0.0127	0.0331	-14.2%
Body mass	-0.0204	0.0130	-21.5%
Sum of Skinfolds	-0.0197	-0.0084	+0.8%
Vertical Jump	-0.0110	0.0124	-1.2%
Medicine Ball Throw	-0.0031	0.0197	+4.0%
10 m Sprint	-0.0094	-0.0256	-6.5%
60 m Sprint	-0.0163	-0.0276	-13.8%
Agility 505 right	-0.0131	-0.0138	-43.0%
Agility 505 left	-0.0127	-0.0257	-9.1%
Estimated VO_2max_	0.0141	0.0015	-4.7%
Attributable variance	37.44%	17.00%	

**Fig 2:**
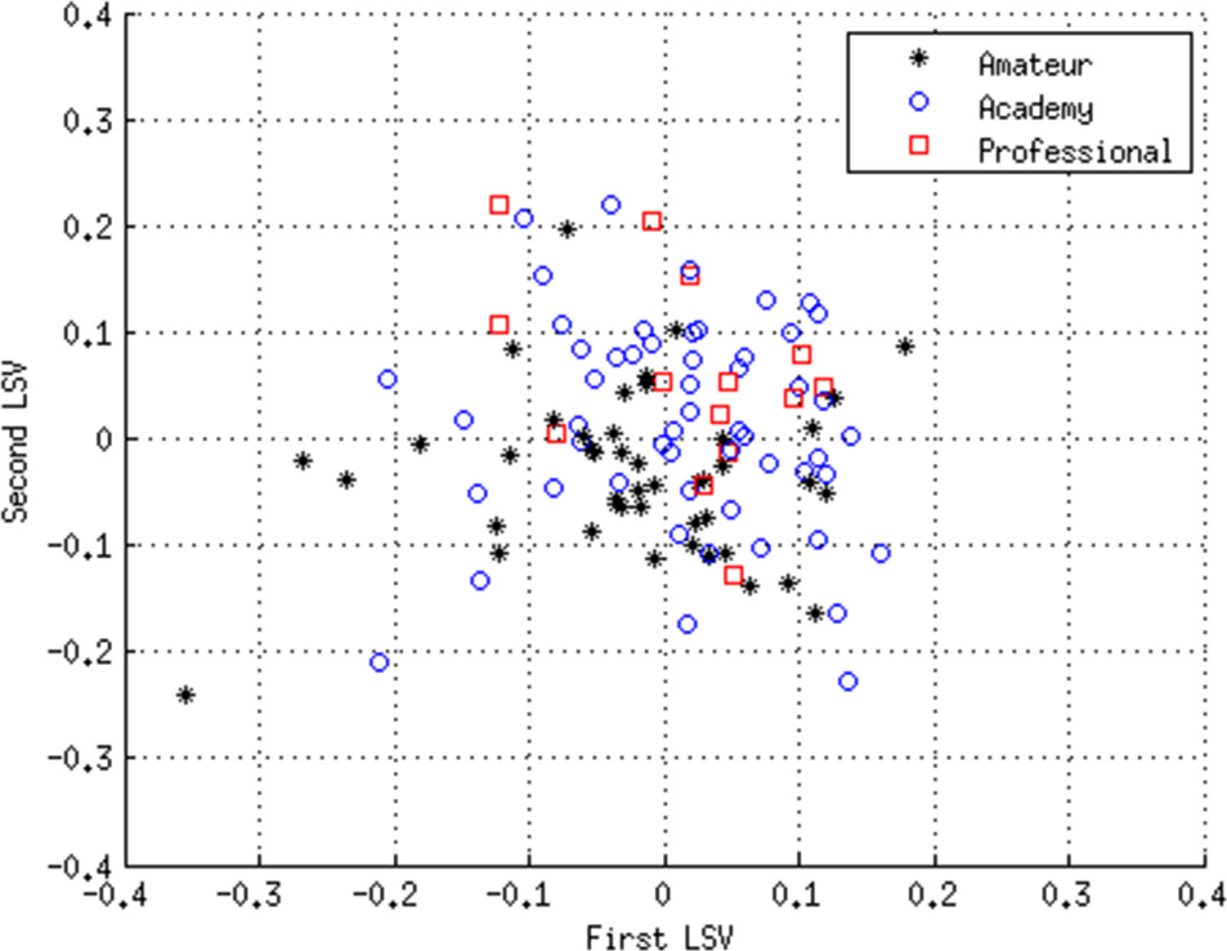
Results of SVD analysis of the exploratory dataset using the variables: Years from PHV; Height; Body Mass, 10 m Sprint; 60 m Sprint; Agility 505 right; Agility 505 left; and Estimated VO_2max_. Scatter-plot of the first and second left singular vectors (LSVs).

Based on the results of the SVD analysis, the sum of skinfolds, vertical jump and medicine ball throw variables were excluded from the model. The SVD analysis was then repeated with the reduced dataset. The results of this new analysis are presented in Fig 2, which shows a scatter-plot of LSV1 and LSV2. From this it can be seen that the professionals tend to cluster in the top half of the plot, while the amateurs mainly occupy the lower half. By comparison the academy players are more widely dispersed, with considerable overlapping occurring between them and the professional group.

Table 4 presents the variable coefficients calculated through SVD analysis of the reduced set of variables. These represent the contribution that each variable makes to the respective LSVs. From Table 4 it can be seen that in the new model, LSV1 now accounts for 41.3% of the variance in the data, while LSV2 accounts for a further 21.8%. As such, the optimized model captures more variance in the first two LSVs than the previous model.

**Table 4:** Variable coefficients derived from SVD analysis of the reduced set of variables in the exploratory dataset.

Variable	LSV1 coefficients	LSV2 coefficients
Years from PHV	-0.0217	0.0300
Height	-0.0175	0.0353
Body mass	-0.0247	0.0164
10 m Sprint	-0.0126	-0.0205
60 m Sprint	-0.0203	-0.0254
Agility 505 right	-0.0158	-0.0227
Agility 505 left	-0.0150	-0.0333
Estimated VO_2max_	0.0163	0.0021
Attributable variance	41.3%	21.8%

**Fig 3:**
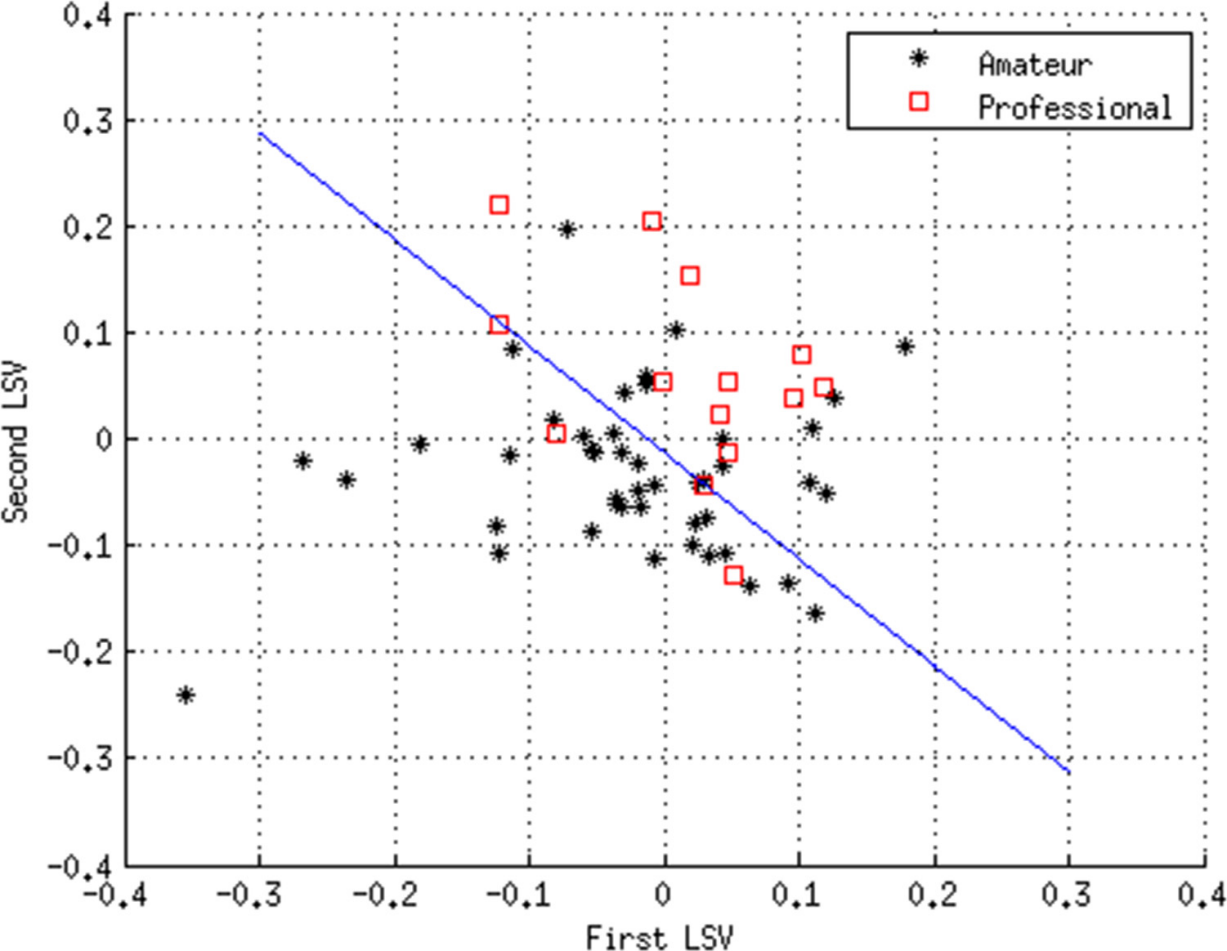
Cut-off demarcation line (blue line) necessary to achieve optimum separation of the amateur and professional sub-groups in the exploratory dataset.

Table 5 shows the results of the ROC analysis using the diagnostic vector produced using the rotated SVD model described above. It can be seen that for the exploratory dataset the model was able to distinguish with a high degree of accuracy (sensitivity = 85.7%, specificity = 71.1%; *p*<0.001) between future amateur and professional cohorts using the variables listed in Table 4. This is illustrated in Fig 3, which shows the cut-off demarcation line necessary for the optimum sensitivity and specificity scores. Table 5 shows that although the model can identify the amateurs from academy (sensitivity = 73.3%, specificity = 68.5%; *p*<0.001) and professional players with reasonable accuracy, it could not distinguish between professional and academy players.

**Table 5:** Results of the ROC analysis using the exploratory dataset.

	Area under curve	Cut-off value	True positives	False negatives	True negatives	False positives	Sensitivity	Specificity	P
Professional vs rest	0.693	0.0226	10	4	59	40	71.4	59.6	0.0097
Amateur vs rest	0.725	-0.0117	32	13	50	18	71.1	73.5	<0.0001
Professional vs amateur	0.800	-0.0118	12	2	32	13	85.7	71.1	<0.0001
Professional vs academy	0.603	n.s.	n.s.	n.s.	n.s.	n.s.	n.s.	n.s.	n.s.
Amateur vs academy	0.706	-0.0044	33	12	37	17	73.3	68.5	<0.0001

n.s.–not significant

### Validation results

Table 6 presents the anthropometric and fitness characteristics of the amateur, academy and professional players for the blinded validation dataset. The ANOVA revealed significant differences between the amateur, academy and professional sub-groups for the vertical jump (*p* = 0.036), medicine ball throw (*p* = 0.011), agility 505 right (*p* = 0.011) and agility 505 left (*p* = 0.001).

**Table 6:** Descriptive statistics and ANOVA results for the amateur, academy and professional sub-groups in the validation dataset.

	Amateurs (n = 34)	Academy (n = 43)	Professional (n = 15)	P
Years from PHV (years)	1.83 ± 0.61	1.84 ± 0.59	1.83 ± 0.34	0.997
Height (cm)	177.6 ± 6.2	178.6 ± 5.9	179.5 ± 4.6	0.525
Body mass (kg)	76.7 ± 13.5	77.5 ± 10.6	75.8 ± 7.3	0.807
Sum of Skinfolds (mm)	43.5 ± 18.9	43.4 ± 16.5	37.2 ± 10.0	0.173
Vertical Jump (cm)	37.3 ± 4.6	39.0 ± 4.9	42.6 ± 6.8	0.036
Medicine Ball Throw (m)	5.7 ± 0.8	5.8 ± 0.7	6.3 ± 0.6	0.011
10 m Sprint (s)	1.88 ± 0.16	1.89 ± 0.16	1.84 ± 0.16	0.637
60 m Sprint (s)	8.22 ± 0.38	8.29 ± 0.41	8.04 ± 0.31	0.082
Agility 505 right (s)	2.46 ± 0.14	2.46 ± 0.13	2.37 ± 0.08	0.011
Agility 505 left (s)	2.52 ± 0.14	2.47 ± 0.12	2.40 ± 0.07	0.001
Estimated VO_2max_ (ml.kg^-1^.min^-1^)	50.1 ± 4.2	49.8 ± 4.2	51.5 ± 4.3	0.468

**Fig 4:**
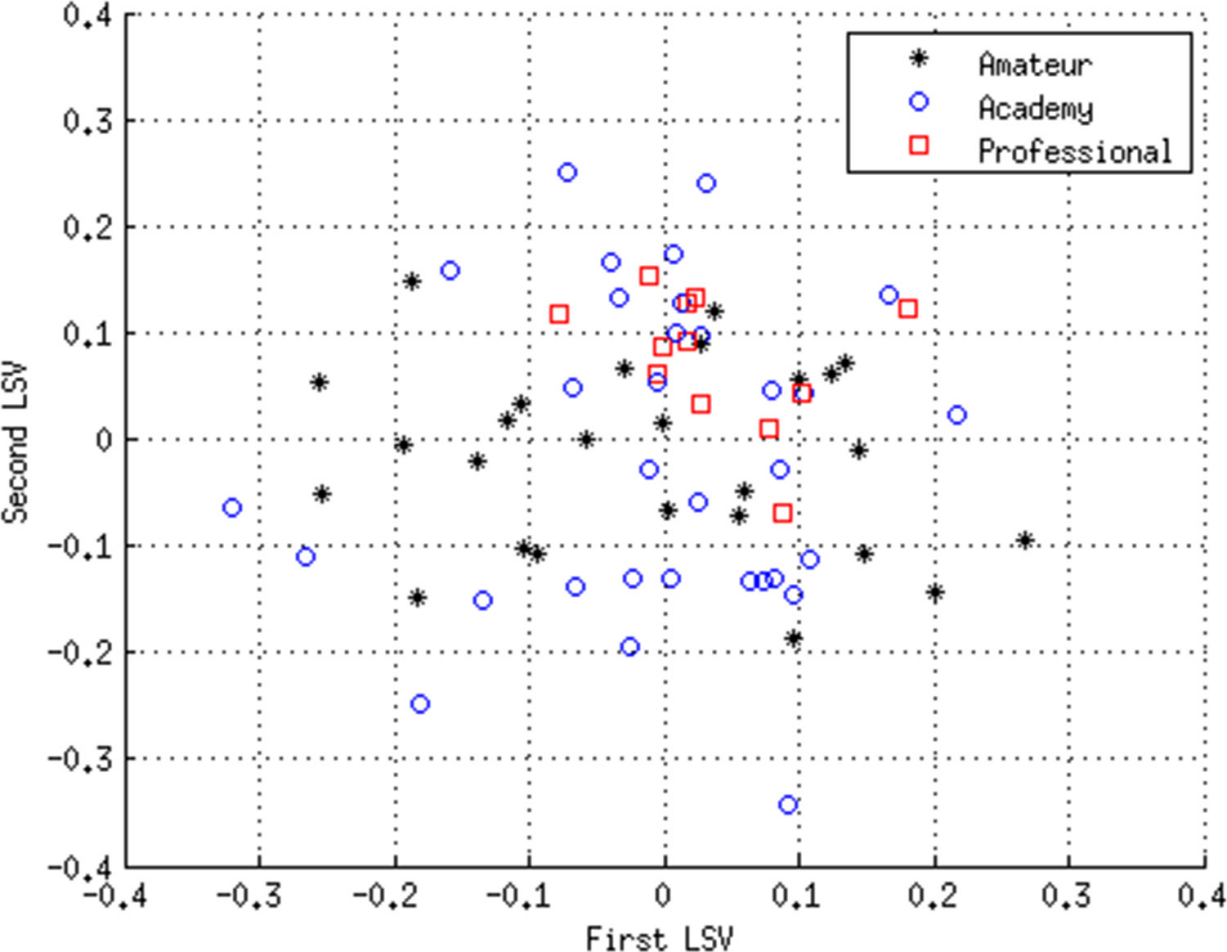
Results of SVD analysis of the validation dataset using the variables: Years from PHV; Height; Body Mass; 10 m Sprint; 60m Sprint; Agility 505 right; Agility 505 left; Estimated VO_2max_. Scatter-plot of the first and second left singular vectors (LSVs).

Fig 4 shows the scatter-plot of LSV1 and LSV2 for the validation dataset using the optimum SVD model developed. It can be seen that, as with the exploratory dataset, the professionals tend to cluster in the top half of the plot. However, unlike the exploratory dataset, the amateurs are much more widely dispersed, as are the academy players.

Table 7 shows the results of the ROC analysis for the SVD model using the validation dataset. The model was able to distinguish with reasonable accuracy the future professionals from the amateur (sensitivity = 83.3%, specificity = 69.2%; p = 0.002) and academy (sensitivity = 100.0%, specificity = 56.3%; p = 0.011) players. However, it was unable to identify the future amateurs from the academy players.

**Table 7:** Results of the ROC analysis using the validation dataset.

	Area under curve	Cut-off value	True positives	False negatives	True negatives	False positives	Sensitivity	Specificity	Significance (p value)
Professional vs rest	0.737	-0.0346	10	2	37	21	83.3	63.8	0.0034
Amateur vs rest	0.591	n.s.	n.s.	n.s.	n.s.	n.s.	n.s.	n.s.	n.s.
Professional vs amateur	0.766	0.0284	10	2	18	8	83.3	69.2	0.0015
Professional ve academy	0.603	-0.0001	12.	0	18	14	100.0	56.3	0.0111
Amateur vs academy	0.525	n.s.	n.s.	n.s.	n.s.	n.s.	n.s.	n.s.	n.s.

n.s.–not significant

Table 8 shows the contributions of the variables towards the respective diagnostic vectors for the exploratory and validation datasets, with each coefficient being ranked according to its absolute value. This reveals that for both the exploratory and validation datasets the 60 m sprint and agility 505 variables occupied the top three ranks, with body mass and maturity occupying the lowest ranks.

**Table 8:** Rotated coefficients of the diagnostic metrics for the exploratory and validation datasets.

Variable	Rotated Coefficients	Coefficient Rank	Rotated Coefficients	Coefficient Rank
	(Exploratory)	(Exploratory)	(Validation)	(Validation)
Years from PHV	0.0058	8	0.0056	8
Height	0.0126	6	0.0234	4
Body mass	-0.0059	7	-0.0100	7
10 m Sprint	-0.0235	4	-0.0169	6
60 m Sprint	-0.0323	2	-0.0404	3
Agility 505 Right	-0.0273	3	-0.0459	1
Agility 505 Left	-0.0342	1	-0.0456	2
Estimated VO_2max_	0.0130	5	0.0187	5

## Discussion

The primary aim of the study was to develop a new higher-dimensional methodology, based on SVD, which could be used to better analyse multivariate datasets and assist in the TID of youth athletes. In order to assess the validity of the new methodology, this was applied to anthropometric and fitness data collected from junior Under 15 rugby league players, with the aim of establishing the extent to which these characteristics could be used to predict long-term career attainment. The study findings showed that future career attainment (i.e., professionals playing in Super League) could be predicted with reasonable accuracy using anthropometric and fitness characteristics. With both the exploratory and validation datasets, the rotated SVD method was able to accurately distinguish most of the professional players from the amateurs (sensitivity >83%) using maturity, height, body mass, 10 and 60 m sprint, agility 505 and estimated $\dot{V}O_{2\text{max}}$. However, the SVD model was less successful at differentiating the academy players from both the amateur and professional players. As such, the findings of the present study support those of previous studies [10, 11] and show that SVD analysis may be an appropriate statistical technique to employ with TID.

Like PCA, to which it is closely related, one of the major advantages of SVD analysis over conventional multivariate statistical techniques is its ability to capture most of the variance in the data in a few composite variables, enabling cluster analysis to be performed and distinctions between sub-groups to be readily identified. In addition, SVD analysis can readily visualize, in 2-D or 3-D, complex higher-dimensional datasets with many variables. By forming the eigensystem of the data, it is possible to orthogonalize the data and identify the most important variables that account for most of the variance in the system. Orthogonalizing the data also avoids multicollinearity problems that are often associated with more standard statistical techniques, such as MANOVA. If the principal LSVs are plotted against each other on a scatter-plot it is possible to visualise the eigensystem of the data, thus enabling a deeper understanding of the underlying relationships within the data. As such, these LSV scatter-plots give a ‘true’ visualization of the relationships within the data; something that is generally not possible using more standard techniques. If one compares Fig 2 with Fig 4 it can be seen that in both figures, the professionals cluster in the top half of the scatter-plot. There is also a reasonable degree of separation between the amateur and academy players in Fig 2, which is completely absent in Fig 4, something that is supported by the ROC results in Tables 5 and 7. This indicates that in the exploratory dataset the amateur and academy players were more dissimilar in their physiological characteristics than their counterparts in the validation dataset. Indeed with the validation dataset, the amateur and academy players were both much more widely dispersed, with considerable overlap between the two groups.

By combining the first and second LSVs and rotating them through 45°, it was possible to develop a single diagnostic vector (see Appendix) that embodied the combined effect of the measured variables and encapsulated most of the variance in the data, thus allowing ROC analysis to be carried out to calculate the sensitivity and specificity scores. Because the LSVs are themselves made up of a series of linear weighted equations, this made it possible using linear algebra (equation 1 below) to calculate the respective ‘rotated’ coefficients (see Appendix) that should be applied to the measured data in order to recreate the diagnostic vector. Consequently, the contribution of each variable towards the overall diagnostic vector could be quantified.

$$DV=(c_{1}\;x\;v_{1})+(c_{2}\;x\;v_{2})+\cdots +(c_{8}\;x\;v_{8})$$

where, *DV* = diagnostic vector; *c*_1_ to…*c*_8_ = rotated coefficients as shown in Table 8 and *v*_1_…*v*_8_ = vectors for the respective variables shown in Table 8.

**Fig 5:**
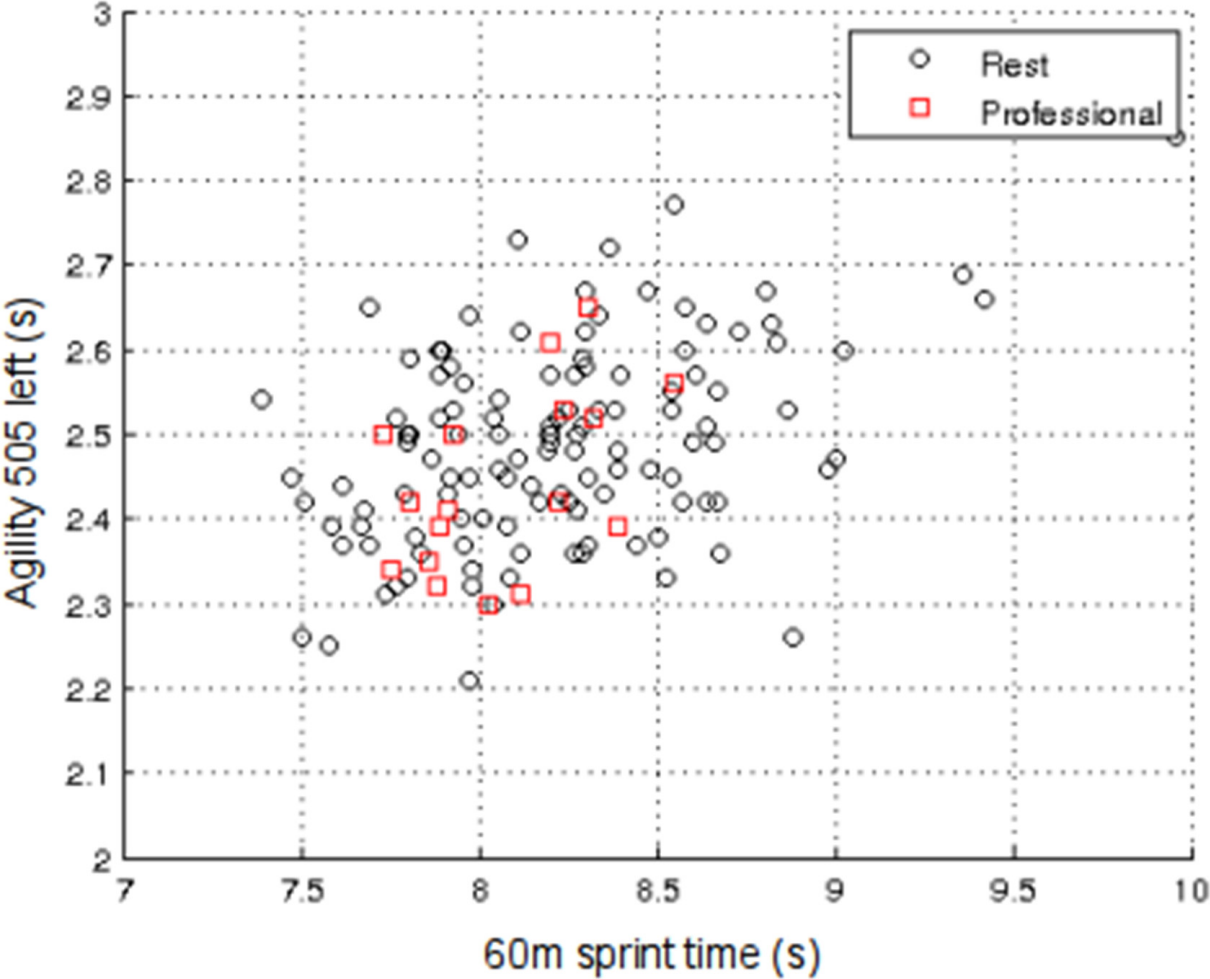
Scatter-plot of 60 m Sprint and Agility 505 left for the exploratory dataset.

**Fig 6:**
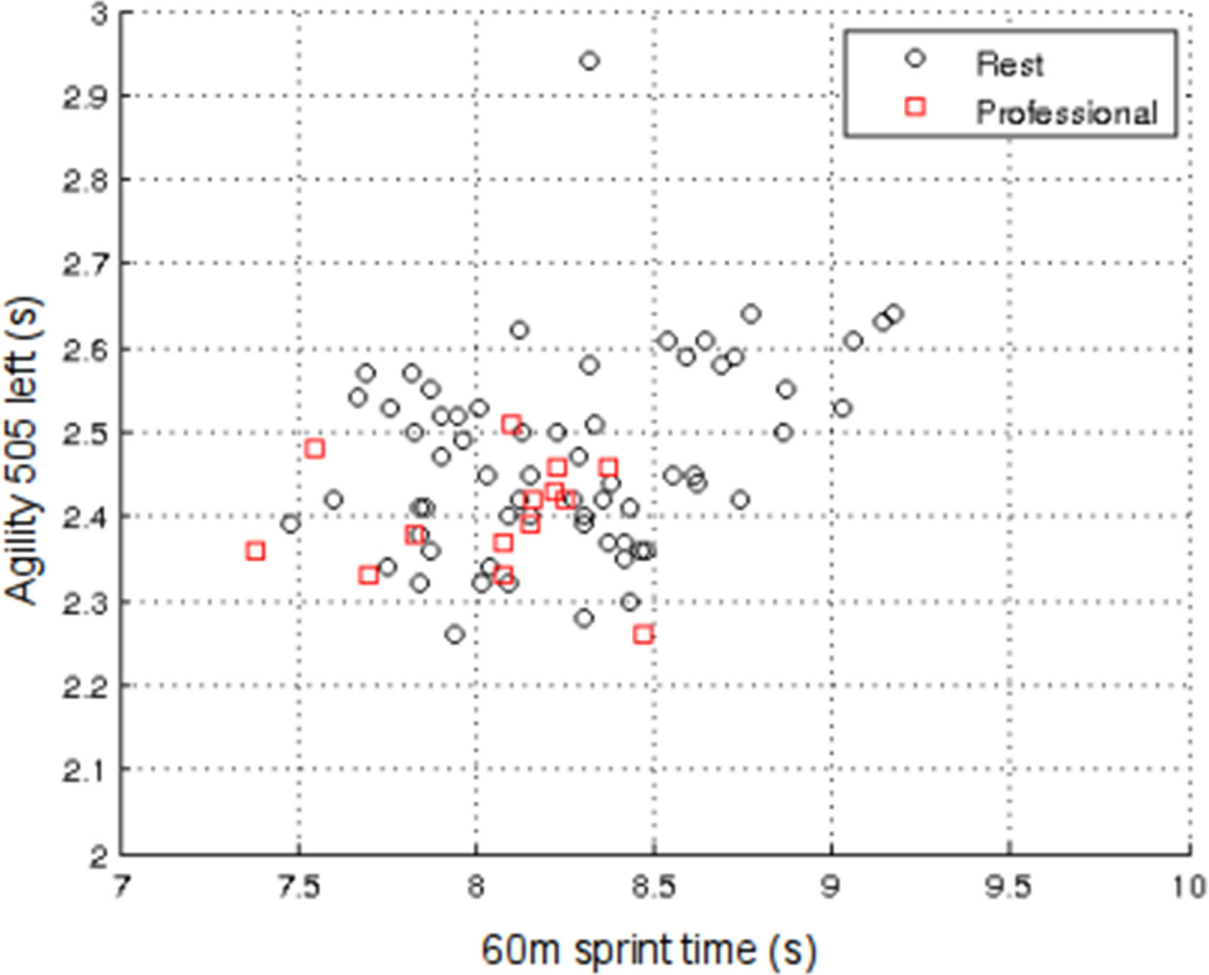
Scatter-plot of 60 m Sprint and Agility 505 left for the validation dataset.

By creating a single diagnostic vector, which was amenable to ROC analysis, we were able to simplify the complexity in the TID datasets and identify those variables that discriminated most between groups of players. The contributions (coefficients) of the variables towards the respective diagnostic vectors for the exploratory and validation datasets are quantified in Table 8. These coefficients reveal that for both the exploratory and validation datasets, 60 m sprint and the agility 505 variables occupy the top three ranks, albeit in different orders for the two datasets. This suggests that these variables are particularly influential when distinguishing junior rugby league players in terms of future career attainment. Figs 5 and 6 show scatter-plots of 60 m sprint and agility 505 left for both the exploratory and validation datasets. These plots mirror those in Figs 2 and 4 and show the professionals clustering towards one end of the distribution. If the variables are combined and rotated through 45° to produce a diagnostic vector, then ROC analysis reveals that it is possible to distinguish the professionals from the rest using just the variables 60m sprint and agility 505 left with sensitivity = 58.8% and specificity = 69.8% (p = 0.044) in the exploratory dataset, and sensitivity = 85.7% and specificity = 52.8% (p = 0.021) in the validation dataset.

While the creation of a diagnostic vector has the advantage that it reduces an inherently complex multivariate system down to a single metric that can be used for TID purposes, it is important to remember that this approach also has limitations. Being a composite metric made up of a linear weighted combination of the measured variables, it is perfectly possible for two athletes to achieve similar diagnostic metric scores and yet exhibit different values for the individual measured variables. Consequently, the overall metric score for a given individual may be adversely influenced by an abnormally high value in one of the key variables, something that could lead to ‘false positive’ result. Having said this, the sensitivity and specificity results achieved at both the exploratory and validation stages suggest that the diagnostic metric was relatively good at predicting those players who would eventually become professionals, suggesting that the variable weightings in the diagnostic vectors had some merit.

In order to simulate an independent follow-on trial, an adopted 'hold out' validation approach, with the randomly selected validation dataset blinded during the development stage of the SVD methodology was used. While this approach simulated an independent follow-up study, it provided no guarantee that the exploratory and validation datasets would be statistically similar. Indeed, inspection of respective datasets revealed noticeable differences between the two. In the exploratory dataset the future amateur players performed better than the professionals in the medicine ball throw, while the situation was reversed in the validation dataset. This suggests that although the medicine ball throw was excluded from the model at the exploratory stage, it may actually be a more important TID indicator than the exploratory results suggest. Furthermore, in the validation dataset the future academy players were slower over 60 m compared with the amateurs, something that was not exhibited in the exploratory dataset. Consequently, it is not surprising that the SVD analysis revealed marked differences between the exploratory and validation studies with regard to the status of the academy players—differences that reflected variations between the two datasets used rather than the SVD methodology itself. Having said this, it is clear from the ROC results that the SVD methodology was only partially successful at TID when using anthropometric and fitness data. While the sensitivity results relating to the future professional players were generally high, the specificity results were much lower, with the SVD model finding it much harder to distinguish between the future academy players and the other two groups. As such, this highlights the limitations of using purely anthropometric and fitness data for TID in young athletes. Many other technical, tactical and psychological factors influence the career outcomes of athletes, making TID a multidimensional problem [9]. Further work will therefore be required to explore the potential of SVD in TID using datasets containing a broader range of variables reflecting technical, tactical and psychological factors, alongside anthropometric and fitness indicators. In particular, the inclusion of broader range of variables in the SVD model may help to explain the presence of the large number of ‘strong false positives’ in the present study. Because developmental changes are also likely to be influential, both pre and post-15 years of age, it is recommended that further studies be undertaken to evaluate the efficacy of the SVD approach using longitudinal data. Given that physiological and psychological changes occur rapidly during adolescence, it is important that the impact of these changes be fully assessed before TID diagnostic criteria are established.

The findings of the present study are consistent with those of previous studies using more conventional statistical techniques [10, 11] and clearly indicate that younger players with greater speed and agility are more likely to progress towards professional status, irrespective of their height, mass and maturational status as measured by PHV. Collectively, the sensitivity and specificity results suggest that players with poor physical qualities at the Under 15 age category are unlikely to progress to professional status, and that those with superior physical qualities have a distinct advantage, something that is clearly evident in both Figs 2 and 4. However, the specificity results also reveal that young players with advanced anthropometric and fitness characteristics are not necessarily guaranteed advanced career outcomes, as can be seen by the number of ‘false positives’. Conversely, a few ‘false negative’ players progress to the highest level despite having less well developed fitness characteristics. So although the study demonstrates that it is possible to objectively identify talent using anthropometric and fitness data for selection and de-selection (exclusion) purposes, there is the potential for ‘false negative’ diagnosis, something that in effect would mean the exclusion of potential professional Super League rugby league players. As such, the results of the present study should be treated with caution, and, in any event, it is unwise to rely on physical data alone for TID purposes. Although higher dimensional models such as the one presented in this study have great potential, the dynamic and complex nature of TID cannot be reconciled from physical data alone [1, 3, 29]. To progress the field of TID, player profiling should be inclusive of psychological, and sociological factors, in addition to attributes specifically aligned to the technical and tactical demands of the sport. Only then is it likely that the sensitivity or specificity will increase for identifying future talent.

## Conclusion

Early age TID of athletes is a complex, multi-dimensional problem, which necessitates the use of a multivariate approach. The higher-dimensional methodology presented in this paper demonstrates that it is possible using SVD to simplify an inherently complex problem in order to determine criteria, with which to identify future professional athletes. By coupling SVD and ROC analyse together it is possible to quickly assess likely outcomes and thus identify appropriate criteria thresholds. As such, this new SVD based methodology appears to have considerable potential as a TID tool.

With respect to TID in youth rugby league players, while the SVD model does not completely eliminate ‘false negative’ removal of potentially talented players, it does demonstrate that by using a multivariate SVD methodology it is possible to differentiate between future professional and amateur players, using anthropometric and fitness characteristics alone with a reasonable degree of precision. As such, the study shows that multivariate higher-dimensional analysis can identify differences between groups, which can be readily visualized, facilitating the identification of variables that are influential in distinguishing between sub-groups. While the new methodology appears to have great potential in TID, care must be taken when considering variables for inclusion in the SVD model. Although the model can distinguish between future amateur and professional rugby league players using anthropometric and fitness data, this type of data appears to be insufficient when trying to distinguish between future academy and professional players. Consequently, technical, tactical and psychological factors should also be considered. Further research will therefore be required to minimise the potential for false positive and false negative diagnoses and also to assess the practical application of the new higher-dimensional methodology in TID.

## Appendix

Singular value decomposition (SVD) was used to visualize the differences between the amateur, academy and professional cohorts. Although closely related to principal component analysis (PCA), SVD was used in preference to PCA because it eliminated the need to generate a covariance matrix, thus making it more computationally efficient. In order to perform SVD on the data, we created a (m x n) matrix, *X*, containing selected data from all three cohorts. The columns of the *X* matrix comprised the variables selected for analysis, which we mean-adjusted and standardized to unit variance, while the rows represented the subjects included in the analysis.

SVD was then performed on *X* as follows:

$$X=U.S.V^{T}$$ (A1)

where *U* is a (m x n) left singular vector (LSV) matrix with identical dimensions to *X*; *S* is a (n x n) diagonal singular value (SV) matrix; and *V* is a (n x n) right singular vector (RSV) matrix. In *U*, the columns (LSVs) are orthogonal composites of the original variables in *X*, with the rows equating to the participants in the study. The SVs are the square-roots of the eigenvalues of the data and as such were used by us to calculate the variance attributable to the respective LSVs. By plotting the first and second LSVs (i.e. the LSVs associated with the first and second eigenvalues) against each other we were able to produce 2-dimensional scatter plots of the orthogonalized data, which captured most of the variance in the system. By identifying the elements of *U* that belonged to the amateur, academy and professional cohorts, respectively, it was then possible to perform cluster analysis.

The respective LSVs comprise linearly weighted combinations of the original variables. We calculated the linear coefficients for each LSV using the Moore-Penrose pseudoinverse algorithm, as follows:

$$Y=(X^{T}.X){}^{\;-1}.X^{T}.U$$ (A2)

We then combined the respective coefficients for the first and second LSVs and rotated them through forty-five degrees, as follows:

$$r=[cv_{1}.\text{cos}(45)]+[cv_{2}.\text{sin}(45)]$$ (A3)

where *r* is a vector containing the rotated combined coefficients and *cv*_*1*_ and *cv*_*2*_ are vectors containing the linear coefficients for the first and second LSVs, respectively. By examining the magnitude of the coefficients in *r* we were able to assess the relative contribution of the respective variables to the variance of the system.

Finally, we combined the first and second LSVs into a single diagnostic vector, *d*, which we rotated through forty-five degrees, as follows:

$$d=[u_{1}.\text{cos}(45)+[u_{2}.\text{sin}(45)]$$ (A4)

where *u*_*1*_ and *u*_*2*_ are the first and second LSVs, respectively.

The diagnostic vector, *d*, was then incorporated into a matrix with a binary outcome classifier (i.e. a vector containing ones and zeros representing the outcome status of the subjects included in the analysis) and exported for receiver operating characteristic (ROC) analysis.

## Supporting Information

S1 File Exploratory Dataset. (XLSX)

S2 File Validation Dataset. (XLSX)
